# Research Progress of Exosome‐Derived microRNA in Alcohol Use Disorders: A Critical Review

**DOI:** 10.1111/adb.70124

**Published:** 2026-01-28

**Authors:** Bo Zhang, Ting ting Xie, Qiang Ma, Ya ping Jiang, Yu mei Wang

**Affiliations:** ^1^ Department of Psychology Shandong Provincial Hospital Affiliated to Shandong First Medical University Jinan Shandong China; ^2^ Karamay Hospital of Integrated Chinese and Western Medicine Karamay Xinjiang China; ^3^ Reproductive and Genetic Department of Tai'an Central Hospital Affiliated to Qingdao University Tai'an China

**Keywords:** alcohol use disorder, biomarker, exosome, microRNA

## Abstract

Alcohol use disorder (AUD) is a chronic, relapsing condition that causes extensive systemic damage, yet clinically actionable biomarkers remain lacking. Exosome‐derived microRNAs (exo‐miRNAs) have emerged as highly stable extracellular indicators of disease state and active regulators of alcohol‐induced pathological processes. Unlike proteins or lipids, miRNAs are selectively packaged, cell‐type specific and mechanistically linked to inflammation, hepatocellular injury, synaptic dysfunction and neuroimmune signalling. Here, we provide an integrated and updated review of how chronic alcohol exposure reshapes exo‐miRNA cargo across organs—particularly the liver, immune system and central nervous system. We summarize the biogenesis and selective sorting of exo‐miRNAs, highlight key candidate miRNAs such as miR‐122, miR‐155, miR‐192, miR‐29a, miR‐30a and miR‐124 and analyse their representative gene targets and downstream effects. Furthermore, we distinguish exo‐miRNAs with diagnostic potential from those representing promising therapeutic targets and discuss major limitations, including specificity relative to other drugs of abuse. By integrating mechanistic and translational evidence, this review aims to clarify the biological and clinical value of exo‐miRNAs and to provide guidance for future precision‐medicine strategies in AUD.

## Graphical abstract

Exosome‐derived microRNAs represent stable and mechanistically active mediators of alcohol‐induced multi‐organ injury. This review summarizes how chronic alcohol exposure reshapes exosomal miRNA cargo across the liver, immune system, and brain, highlighting key miRNAs with diagnostic value and therapeutic potential. These findings support exosomal miRNAs as promising tools for precision medicine in alcohol use disorder.

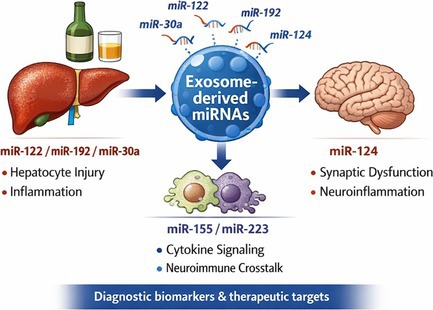

## 1 Introduction

Alcohol use disorder (AUD) significantly impacts both the physical and mental health of patients and is driven by a combination of genetic and environmental factors [1], with genetic factors contributing approximately 30%–70% of disease risk [2]. According to the latest World Health Organization data (2024), approximately 2.6 billion people consume alcohol worldwide, and 400 million meet criteria for AUD, resulting in 2.84 million deaths annually—about 5% of all global deaths [3]. The economic burden is equally staggering: in the United States alone, excessive alcohol consumption incurs more than 249 billion US dollars each year in healthcare expenditures, lost productivity and criminal justice costs [4]. The pathophysiology of AUD involves complex interactions among genetic susceptibility (accounting for 40%–60% of risk variance), epigenetic modifications, neuroadaptive changes, disruption of the gut–brain axis and environmental exposures [5, 6]. Notably, single‐cell RNA sequencing studies have revealed cell‐type‐specific transcriptional signatures in both brain and peripheral tissues of individuals with AUD [7], indicating that different cell populations exhibit distinct molecular responses to chronic alcohol exposure. Despite these advances in mechanistic understanding, clinical management of AUD remains profoundly challenging. Diagnostic practice still relies mainly on structured interviews and self‐report instruments, and objective biomarkers for disease staging, treatment response monitoring or relapse prediction are lacking. Pharmacological treatment options are limited to three FDA‐approved medications (disulfiram, naltrexone and acamprosate), which show only modest efficacy and considerable interindividual variability in response [8]. This therapeutic gap is particularly concerning because untreated AUD follows a chronic, relapsing course with cumulative organ damage and progressive neurodegeneration.

miRNAs play essential regulatory roles in gene expression during brain development [9] and are involved in central nervous system (CNS) formation [10]. Numerous studies indicate that genes involved in neural development are critically implicated in the aetiology of psychiatric disorders [11], and genome‐wide association studies suggest that miRNAs make substantial contributions to neurodevelopmental risk in these conditions [12]. miRNAs participate in multiple processes, including brain development [13], synapse formation [14], synaptic plasticity [15] and neuroimmune signalling [16]. Recent work further demonstrates that manipulating miRNAs can modulate addiction‐related behaviours [17].

Exosomes—nanosized extracellular vesicles enriched in proteins, lipids and nucleic acids—support long‐distance intercellular communication and protect miRNAs from degradation [18, 19, 20]. Importantly, exosomes can cross the blood–brain barrier [21], making them particularly valuable for interrogating CNS changes in AUD. Collectively, these observations suggest that exosome‐derived miRNAs could serve as powerful diagnostic and therapeutic tools in alcohol and other substance use disorders.

However, research on exo‐miRNAs in AUD remains fragmented. Few reviews have systematically integrated their biogenesis, organ‐specific signatures, diagnostic performance and therapeutic utility. To address this gap, we clarify the advantages of exo‐miRNAs over other exosomal cargos, summarize alcohol‐altered exo‐miRNAs and their mechanistic targets, distinguish diagnostic versus therapeutic applications and highlight key limitations and future directions.

## 2 Why Exosome‐Derived microRNAs? Advantages Over Proteins and Lipids

Exosomes are cell‐derived vesicles that act as messengers, regulating cellular processes by transporting ‘cargo’ between cells [22, 23]. Their protein and nucleic‐acid composition reflects the functional state of the producing cells [24]. Because they are readily detected in biological fluids such as serum, plasma and saliva, exosomes are often described as ‘liquid biopsies’ and are attractive candidates for disease biomarker development [25, 26].

Free RNA is rapidly degraded in circulation, whereas miRNAs encapsulated in exosomes are protected from ribonucleases and thus represent particularly stable biomarkers. Numerous studies have reported the diagnostic value of exosomal miRNAs in neurological and psychiatric disorders [27].

Beyond passive reflection of disease status, exosome‐derived miRNAs exert direct regulatory control over alcohol‐related pathogenic pathways. Several alcohol‐induced exo‐miRNAs modulate inflammatory signalling, hepatocellular injury, oxidative stress, mitochondrial dysfunction and neuroimmune communication [28]. These properties underscore a major mechanistic advantage of miRNAs over exosomal proteins or lipids: Proteins and lipids typically represent downstream phenotypic consequences, whereas miRNAs occupy upstream regulatory positions and directly control expression of key pathogenic genes. This dual role—as sensitive indicators and active effectors—makes exo‐miRNAs uniquely suited as both biomarkers and therapeutic targets in AUD.

## 3 Biogenesis and Alcohol‐Induced Modulation of Exosome‐Derived miRNAs

Exosome biogenesis depends on sophisticated molecular machinery. The canonical endosomal sorting complex required for transport (ESCRT) pathway plays a central role, but ESCRT‐independent mechanisms—mediated by ceramide, tetraspanins and the syndecan–syntenin–ALIX axis—also contribute to intraluminal vesicle formation and exosome release [29].

miRNA loading into exosomes is a highly selective, regulated process rather than passive bulk export. Recent work has identified several RNA‐binding proteins (RBPs) that orchestrate miRNA sorting by recognizing specific sequence motifs and/or promoting condensate formation that enriches defined miRNAs in budding vesicles. Examples include hnRNPA2B1 (which binds a GGAG motif), SYNCRIP/hnRNPQ, and Y‐box protein 1 (YBX1) [30].

Chronic alcohol exposure perturbs this machinery at multiple levels. In hepatocytes and microglia, ethanol upregulates several Rab GTPases (e.g., Rab5, Rab11, Rab27a and Rab35) and alters VAMP and syntaxin family SNARE proteins, in parallel with oxidative and endoplasmic reticulum stress responses that together enhance exosome biogenesis and release [31, 32]. miR‐192 has been identified as an upstream regulator that modulates Rab expression and thereby promotes exosome production in alcohol‐exposed hepatocytes [31].

Alcohol also exerts complex immunomodulatory effects, altering extracellular vesicle (EV) miRNA content and contributing to the pathogenesis of alcohol‐induced organ injury [33]. By reshaping immune signalling, alcohol changes cytokine levels, and these cytokines themselves can be packaged into exosomes and delivered to distant target cells [34].

Proteomics and lipidomics analyses further reveal that exosomes from individuals with AUD exhibit distinct molecular signatures beyond miRNAs, including enrichment of inflammatory proteins (such as HMGB1 and HSP70), altered lipid profiles (increased ceramide and decreased phosphatidylcholine) and enhanced posttranslational modifications such as SUMOylation and ubiquitination [32].

Functional studies demonstrate that exosomes serve as critical mediators of intercellular communication in AUD pathogenesis. Exosomes derived from alcohol‐exposed hepatocytes can transfer pro‐inflammatory miRNAs to naïve cells, propagating cellular stress responses [35]. Similarly, neuronal and glial exosomes carrying alcohol‐induced miRNA signatures can activate microglia, contributing to neuroinflammation and synaptic pruning [36]. This intercellular dissemination of pathological signals via exosomes constitutes a novel mechanism of disease progression and provides multiple potential therapeutic entry points. Taken together, these findings highlight exosome‐derived miRNAs as promising levers for precision intervention in AUD.

## 4 Exosome‐Derived miRNAs as Diagnostic Biomarkers in Alcohol‐Associated Liver Disease (ALD)

ALD remains the leading cause of liver‐related mortality worldwide, spanning a spectrum from simple steatosis to alcoholic hepatitis, cirrhosis and hepatocellular carcinoma. Recent single‐cell transcriptomic analyses have shown that alcohol induces cell‐type‐specific molecular responses in hepatocytes, Kupffer cells and hepatic stellate cells, each displaying distinct exosomal miRNA release profiles that collectively shape disease progression [37].

Alcohol exposure also remodels exosome biogenesis by altering the expression of regulators involved in vesicle formation and trafficking. Mechanistically, ethanol modulates Rab GTPases, VAMP proteins and syntaxins—partly via miR‐192—leading to increased exosome production and release [31]. Converging evidence indicates that four major cellular processes are disrupted during ethanol exposure—autophagy, endoplasmic reticulum stress, oxidative stress and epigenetic regulation—which together drive ALD pathogenesis [28].

Within this context, exosomal miRNAs have emerged as highly sensitive, organ‐enriched biomarkers capable of detecting alcohol‐induced injury with superior precision compared with traditional serum markers. Their encapsulation within the exosomal membrane allows them to persist in the circulation while preserving tissue‐of‐origin information, enabling more accurate mapping of alcohol‐induced liver damage.

Organ‐specific exosome profiling has identified several exo‐miRNAs with strong diagnostic performance. In multiple models of liver injury, circulating miR‐122—including exosome‐associated fractions—rises earlier and more robustly than alanine aminotransferase (ALT) and correlates closely with histopathological severity, indicating higher sensitivity for detecting liver damage [38]. In patients with alcoholic hepatitis, the number of circulating exosomes and their miR‐122 cargo is significantly increased and associates with disease activity [39]. Receiver operating characteristic (ROC) analyses further demonstrate good diagnostic accuracy for miR‐192, miR‐122 and miR‐30a in distinguishing alcohol‐induced liver injury [39].

Among the exo‐miRNAs implicated in ALD, the miR‐155/miR‐122 axis has emerged as a central regulator of alcohol‐induced liver inflammation and fibrosis.

miR‐122 is a hepatocyte‐specific, highly abundant miRNA, whose circulating levels are significantly correlated with ALT elevations across multiple models of liver injury, and which is predominantly released in an exosome‐associated form during alcoholic and inflammatory liver damage [40]. Exosomes released from ethanol‐treated hepatocytes can horizontally transfer miR‐122 to circulating monocytes, sensitizing them to LPS stimulation and exacerbating systemic inflammation [35].

miR‐192 is upregulated in alcohol‐exposed hepatocytes and regulates Rab27a and related vesicular trafficking factors, thereby promoting exosome biogenesis and altering exo‐miRNA cargo [31].

Patients with progressive liver disease (cirrhosis) show higher EV‐associated miR‐21 levels, which have been proposed as a marker of fibrosis progression [41].

Depletion of miR‐21 may reduce cytokine production in hepatic stellate cells and impair macrophage chemotaxis during alcoholic liver injury, suggesting that targeting miR‐21 holds therapeutic potential for preventing ALD progression [42].

miR‐223, primarily released by neutrophils, serves an anti‐inflammatory role by targeting IL‐6, and its deficiency in ethanol‐fed models leads to excessive neutrophil infiltration, oxidative stress and aggravated liver injury [43]. Conversely, delivery of exosomal miR‐223 to hepatocytes exerts hepatoprotective effects by dampening inflammatory signalling [44].

**FIGURE 1:**
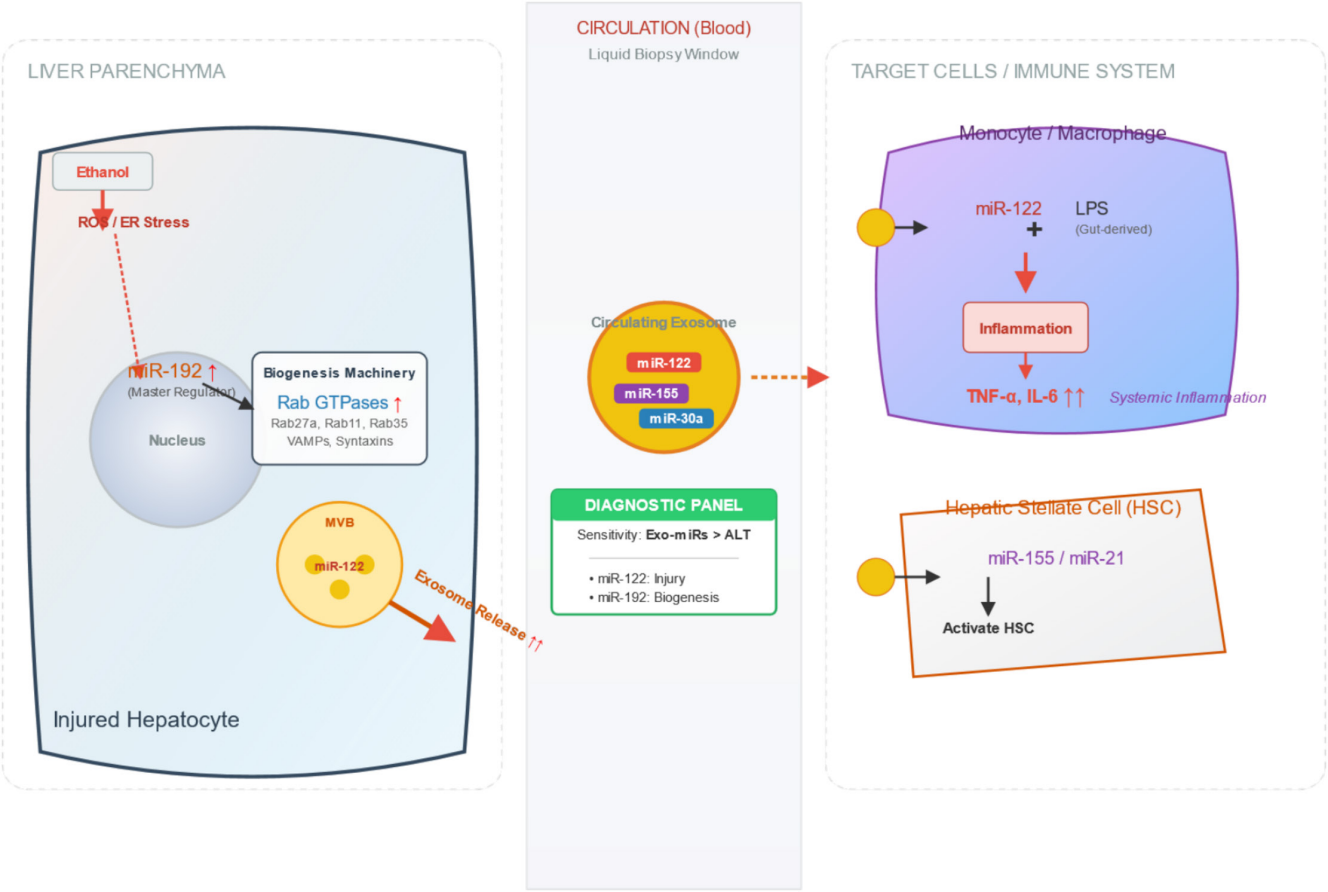
Exosome‐derived miRNAs in alcohol‐associated liver disease (ALD).

Taken together, these miRNAs reflect a complex, organ‐spanning network of exosome‐mediated regulatory signals that drive inflammation, fibrosis and cell death in AUD. Their tissue specificity, upstream positioning in pathogenic cascades and crosstalk between immune, hepatic and neuronal cells underscore their potential as both mechanistic targets and circulating biomarkers for alcohol‐induced injury. To provide a concise overview, Figure 1 summarizes the main sources, representative targets, mechanisms and pathological roles of key exosome‐derived miRNAs implicated in AUD.

## 5 Therapeutic Targeting of the Exosome–miRNA axis in AUD

Accumulating evidence indicates that exosome–miRNA signalling is not merely a biomarker of alcohol‐induced injury, but an active driver of hepatotoxicity, immune dysregulation and CNS pathology. Consequently, therapeutic strategies that modulate this axis are gaining increasing attention. Conceptually, these approaches can be grouped into four categories: inhibition of pathogenic miRNAs, replacement of protective miRNAs, engineering of exosome‐based delivery systems and modulation of exosome biogenesis.

### 5.1 Inhibition of Pathogenic miRNAs

One major strategy aims to inhibit miRNAs that are consistently upregulated and causally implicated in AUD pathology. miR‐155 is a prime example: it promotes robust pro‐inflammatory and profibrotic responses in alcohol‐associated liver injury, and anti‐miR‐155 interventions in preclinical models attenuate steatohepatitis and fibrosis [45]. These findings provide a mechanistic basis for developing anti‐miR‐155‐based therapies in AUD‐related liver disease.

### 5.2 Replacement of Protective miRNAs

Conversely, miRNA replacement therapy seeks to restore homeostatic regulation by supplementing protective miRNAs that are downregulated during chronic alcohol exposure. Mesenchymal stem cell (MSC)–derived exosomes enriched with miR‐124 exhibit antifibrotic, anti‐inflammatory and tissue‐repair properties in liver fibrosis models [46], offering direct experimental support for exosome‐mediated restoration of beneficial miRNAs. In the CNS, delivery of miR‐124—either directly or via exosomes—can reprogram microglia toward an anti‐inflammatory phenotype, reduce neuronal apoptosis and support neurogenesis [47]. Together, these results highlight miR‐124 as a cross‐organ protective miRNA with both hepatic and neuroprotective potential.

### 5.3 Engineered Exosomes as miRNA Delivery Platforms

Engineered exosomes further expand the therapeutic landscape by serving as versatile platforms for targeted RNA delivery. MSC‐derived exosomes possess intrinsic regenerative and immunomodulatory properties and can be modified on their surface with neuron‐targeting ligands, such as the rabies virus glycoprotein (RVG) peptide, to enable precise delivery of therapeutic miRNAs across the blood–brain barrier. RVG‐modified exosomes carrying miR‐124 have been shown to efficiently enter the CNS, suppress microglial activation, attenuate neuroinflammation and promote neuronal survival and neurogenesis [47], underscoring the feasibility of applying similar strategies to AUD‐related neurobiological injury.

Compared with traditional lipid‐ or polymer‐based nanoparticles, exosome‐based delivery of synthetic miRNA mimics offers superior biocompatibility, lower immunogenicity, enhanced resistance to nuclease degradation and improved tissue specificity [48]. These advantages make exosome‐based platforms particularly attractive for chronic conditions such as AUD, where repeated dosing may be required.

### 5.4 Modulation of Exosome Biogenesis and Release

A complementary therapeutic avenue targets the upstream machinery that governs exosome production and release. Chronic ethanol exposure disrupts exosome biogenesis by upregulating key vesicular trafficking regulators, including Rab27a, VAMP proteins and syntaxins, thereby markedly increasing exosome output [31]. In line with this, pharmacological inhibition of neutral sphingomyelinase 2 (nSMase2) or genetic silencing of ESCRT‐associated components reduces exosome release under both basal and ethanol‐stimulated conditions [49]. These findings identify nSMase2, Rab27a and related factors as critical regulatory nodes in exosome secretion [50]; inhibiting these pathways can significantly diminish the dissemination of pro‐inflammatory and profibrotic exosomal cargo.

In parallel, delivery of synthetic miRNA mimics has emerged as a promising strategy to counteract alcohol‐related neuroinflammation and multiorgan injury. Administration of miR‐124 mimics—either directly or via engineered exosomes—suppresses microglial activation, mitigates excitotoxic and endoplasmic reticulum‐stress–mediated neuronal injury, and stabilizes synaptic function, demonstrating robust neuroprotective and cross‐organ therapeutic potential [51, 52].

Taken together, these therapeutic strategies—miRNA inhibition, miRNA replacement, engineered exosome‐mediated delivery and modulation of exosome biogenesis—underscore the versatility and promise of targeting the exosome–miRNA axis. As understanding of exosomal communication deepens, these approaches may ultimately form the foundation of precision therapeutics for AUD, providing targeted interventions that address both hepatic and neurobiological components of the disease.

## 6 Conclusion

Exosome‐derived miRNAs occupy central positions in both systemic and CNS pathology associated with AUD. They regulate inflammatory signalling, hepatocellular injury, synaptic remodelling and neuroimmune communication, making them compelling candidates for future diagnostic and therapeutic development. Early studies have demonstrated encouraging diagnostic accuracy and mechanistic relevance, but further work is needed to determine their specificity across different substances of abuse, to validate large‐scale exo‐miRNA diagnostic panels and to optimize safe and effective therapeutic delivery systems.

In the long term, a deeper understanding of the exosome–miRNA axis is expected to drive more precise phenotyping and individualized treatment of AUD, ultimately helping to reduce the substantial global burden of alcohol‐related disease.

## Author Contributions

Bo Zhang designed and wrote the manuscript independently; Qiang Ma and Ya ping Jiang collected literature. Yu mei Wang revised the manuscript. Ting Ting Xie participated in the literature collection, the development of the initial manuscript draft and subsequent revisions.

## Funding

The authors have nothing to report.

## Consent

The authors have nothing to report.

## Conflicts of Interest

The authors declare no conflicts of interest.

## Data Availability

Research data are not shared.
